# Neutrophil extracellular traps in tumor microenvironment for radiotherapy: friend or foe?

**DOI:** 10.3389/fimmu.2026.1638314

**Published:** 2026-05-28

**Authors:** Xuan Luo, Xin Qi, Xiaojia Chang, Zhicheng Wang, Rongrong Liu, Hongguang Zhao

**Affiliations:** 1 Department of Nuclear Medicine, The First Hospital of Jilin University, Changchun, China; 2 National Health Commission (NHC) Key Laboratory of Radiobiology, School of Public Health, Jilin University, Changchun, China; 3 School of Public Health, Jilin University, Changchun, China

**Keywords:** cancer, NETosis, neutrophil extracellular traps, radiotherapy, tumor microenvironment

## Abstract

Neutrophil extracellular traps (NETs) are chromatin-protein complexes released by neutrophils that have dual roles in the tumor microenvironment (TME), participating in anti-tumor immunity and driving malignant progression by promoting tumor proliferation, metastasis, and immune escape. Radiotherapy (RT) exerts anticancer effects by inducing DNA damage and microenvironmental remodeling, but the inflammatory response it triggers can activate the formation of NETs, which exacerbate radioresistance by degrading the extracellular matrix, encapsulating circulating tumor cells, inhibiting CD8+ T-cell infiltration, and promoting distant tumor metastasis. Meanwhile, NETs may act in concert with neutrophils to indirectly enhance radiotherapy efficacy by releasing reactive oxygen species (ROS) and promoting activation and recruitment of tumor-specific T cells. Hypoxia, inflammatory factors, damage-associated molecular patterns (DAMPs), and microbial metabolites in the TME affect radiotherapy outcomes by regulating NET formation. Targeting NETs in combination with radiotherapy reverses immunosuppression and enhances anti-tumoral effects. The functional heterogeneity of NETs subtypes and their spatial and temporal dynamics need to be analyzed in order to optimize individualized treatment strategies.

## 1 Introduction

Radiotherapy (RT) is the preferred anti-cancer modality for more than half of cancer patients, either as a standalone treatment or in conjunction with other modalities such as surgery, chemotherapy, immunotherapy, and targeted therapies. It offers the advantage of local tumor control with relatively few systemic side effects (1). RT is utilized to treat various types of cancer and serves as a complementary therapy to prevent cancer recurrence, as well as a palliative treatment in advanced stages of the disease (2). RT can be administered through an external beam using X-rays, gamma rays, or photons, or it can be delivered internally by implanting a radiation source into or near the tumor (brachytherapy) or via untargeted ^131^I or targeted ^177^Lu-PSMA radioisotopes (3). Despite the notable successes of RT in tumor management, it faces numerous challenges, including the complexity of the tumor microenvironment (TME), the radioresistance of tumor cells, and the issue of radiation-induced damage to normal tissues (4–7). Consequently, an in-depth examination of various factors within the TME and their interactions with radiotherapy is crucial for enhancing the efficacy and safety of this treatment. Radiation-induced tissue damage and acute inflammation lead to the infiltration of neutrophils into tumors.

Neutrophils are typically present in the TME of most solid tumors and are often referred to as tumor-associated neutrophils, which can exhibit either anti-tumoral (N1) or pro-tumoral (N2) effects (8). In clinical settings, some studies have indicated that neutrophils are essential for enhancing RT efficacy (9–11). Conversely, other studies have suggested that neutrophils may promote cellular resistance to RT, implying that they possess pro-tumoral functions (4, 12). Consequently, neutrophils are increasingly recognized as significant contributors to tumor progression, with one key mechanism being the formation of neutrophil extracellular traps (NETs), also known as NETosis. NETs are currently defined as a network of DNA-histone complexes and cytoplasmic and granular proteins such as calreticulin, myeloperoxidase (MPO), and neutrophil elastase (NE). In addition to their role in pathogen defense, NETs may also be implicated in non-infectious pathologies such as obesity (13), type II diabetes (14), atherosclerosis and thrombosis (15), psoriasis (16), systemic lupus erythematosus (17), rheumatoid arthritis (18), and tumors (19–22). In the field of oncology, while the effects of NETs have been studied in various aspects of tumorigenesis, progression, and metastasis, their impact in the context of cancer-related therapies, particularly RT, remains underexplored. This gap motivates our investigation into the potential role of NETs in tumor responses to radiation.

In this review, we will provide a detailed exploration of the formation mechanisms, components, and properties of NETs, along with their roles in the TME. Additionally, we will analyze the influence of the TME on NET formation, the mechanisms by which NETs affect tumor radiotherapy, and the prospects for their clinical application. Through a systematic elaboration of these topics, we aim to offer new perspectives and research directions in the field of radiotherapy, thereby providing a theoretical basis for enhancing the effectiveness and safety of radiotherapy.

## 2 NETs in the TME: mechanisms and general roles

### 2.1 Mechanisms of NET formation

Neutrophil extracellular traps (NETs) are chromatin–protein complexes released by activated neutrophils. Two principal modes have been identified: suicidal NETosis, a form of programmed cell death, and vital NETosis, which preserves neutrophil viability (23, 24). The phenomenon was first observed by Takei et al. (25) and later characterized and named “NETosis” by Brinkmann et al. (26).

Suicidal NETosis is triggered by stimuli such as phorbol esters, immune complexes, or cytokines. It involves NADPH oxidase (NOX)-dependent generation of reactive oxygen species (ROS), which promotes the release of neutrophil elastase (NE) and myeloperoxidase (MPO) from granules. NE and MPO translocate to the nucleus, where NE degrades histones and MPO facilitates chromatin decondensation (27, 28). Concurrently, Ca²^+^ activates peptidylarginine deiminase 4 (PAD4), which citrullinates histones to reduce their affinity for DNA (29, 30). Nuclear and granule membranes disintegrate, and the decondensed chromatin mixed with granular proteins is extruded through gasdermin D−mediated membrane pores (31, 32). A NOX−independent pathway mediated by mitochondrial ROS has also been described (33–36).

Vital NET formation occurs rapidly (5–60 minutes) and can involve either nuclear DNA or mitochondrial DNA. Nuclear DNA release is triggered by lipopolysaccharide (LPS) binding to Toll−like receptor 4 (TLR4) on platelets, through a NOX−independent mechanism requiring TLR2 and complement receptor 3 (24, 37, 38). This process is accompanied by nuclear membrane vesiculation and disruption (37, 39, 40). Mitochondrial DNA release requires granulocyte−macrophage colony−stimulating factor (GM−CSF) together with LPS or C5a and is dependent on mitochondrial ROS and NOX activity (41).

**Figure 1:**
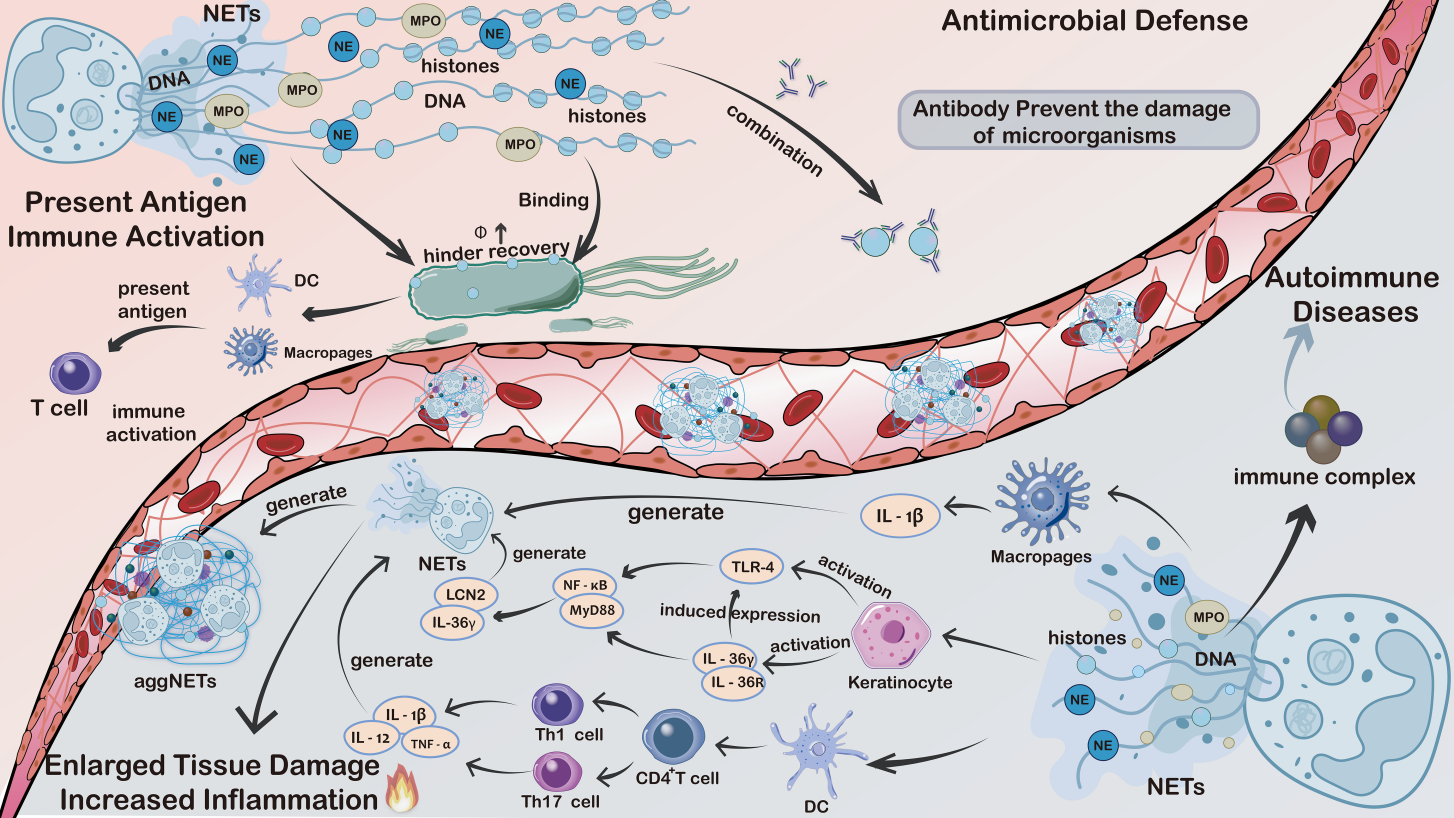
The dual role of NETs. The dual role of neutrophil extracellular traps (NETs). NETs play a beneficial role in innate immunity by trapping and killing pathogens, promoting antigen presentation, and activating immune cells to maintain homeostasis. Conversely, excessive or persistent NETs can have detrimental effects, contributing to the pathogenesis of autoimmune diseases, promoting thrombosis and vascular damage, and exacerbating tissue injury and inflammation.

While NETs play beneficial roles in innate immunity by trapping pathogens, excessive NET formation contributes to pathological conditions including autoimmune diseases and thrombosis (**Figure 1**). In the tumor microenvironment (TME), tumor cells induce NET formation through various mediators. Interleukin−8 (IL−8) binds to CXCR1/2 on neutrophils, promoting NETosis (42–44); IL−8 levels correlate with NET presence in bladder cancer, non−small cell lung cancer (NSCLC), and melanoma (45). Granulocyte colony−stimulating factor (G−CSF) increases neutrophil counts and triggers NETosis (46, 47). Tumor−secreted cathepsin C (CTSC) enhances breast−to−lung metastasis by promoting neutrophil recruitment and NET formation (48). Additionally, NETs encapsulate tumor cells, forming a physical barrier that shields them from CD8^+^ T cells and natural killer (NK) cells, thereby facilitating immune evasion and metastasis (42). Collectively, NETs predominantly exert pro−tumoral effects in the TME, as detailed below.

### 2.2 Promotion of tumor cell proliferation

NETs directly and indirectly promote tumor cell proliferation through multiple signaling pathways. In lung cancer, NE internalized by tumor cells degrades insulin receptor substrate−1 (IRS−1), enhancing PI3K/Akt signaling and driving proliferation (49). NETs also activate TLR9−dependent MAP kinase and NF−κB pathways in various cancers, promoting cell cycle progression (50–53). In colorectal cancer, NETs upregulate MMP9 and activate FAK signaling (54). In diffuse large B−cell lymphoma, IL−8 induces NETs that activate TLR9, NF−κB, STAT3, and p38 pathways (55). Moreover, NETs induce mitochondrial biogenesis via the TLR4−p38−PGC−1α axis, increasing ATP production to fuel tumor growth (56). Beyond direct effects on cancer cells, NETs alter the TME by promoting angiogenesis: in gastric cancer, NETs stimulate CCDC25 translocation in endothelial cells, activating Akt/mTOR signaling and enhancing neoangiogenesis (57). NET−derived extracellular DNA also activates pancreatic stellate cells, promoting fibrotic stroma formation that supports tumor growth (58).

### 2.3 Facilitation of tumor cell migration, invasion and metastasis

NETs promote tumor metastasis through multiple interconnected mechanisms. First, they entrap circulating tumor cells (CTCs) in a β1−integrin−dependent manner, with platelets assisting to form a physical barrier that shields CTCs from CD8^+^ T cells and NK cells (42, 59). Surgical stress enhances this process, and platelet depletion inhibits distant metastasis (60). Second, NETs compromise vascular integrity by disrupting endothelial tight junctions. They bind tumor cells to the vessel wall via von Willebrand factor (VWF) and increase vascular permeability, facilitating extravasation and micrometastasis formation (61, 62). Third, the DNA backbone of NETs serves as a scaffold for proteases including NE, MMP9, and cathepsin G (CG), which degrade ECM components such as laminin and thrombospondin−1 (TSP−1), thereby disrupting tissue barriers and promoting invasion (20, 21). Laminin degradation generates epitopes that activate integrin β1 signaling, while TSP−1 degradation eliminates its metastasis−suppressive effects (21, 48). DNase I treatment or MMP inhibition reduces NET−mediated invasion (63, 64). Fourth, NETs can awaken dormant cancer cells: NE and MMP9 cleave laminin to expose integrin α3β1 binding sites, activating the FAK/ERK/MLCK/YAP signaling pathway and driving proliferative reactivation (21). The NET DNA backbone acts as a scaffold for this process, and DNase I or NE/MMP9 inhibitors prevent recurrence induced by LPS or smoking (21, 65). Degradation of TSP−1 further removes dormancy−maintaining signals (21, 65). Collectively, these mechanisms establish NETs as critical facilitators of metastatic progression.

### 2.4 Regulation of the tumor immune microenvironment

**Figure 2:**
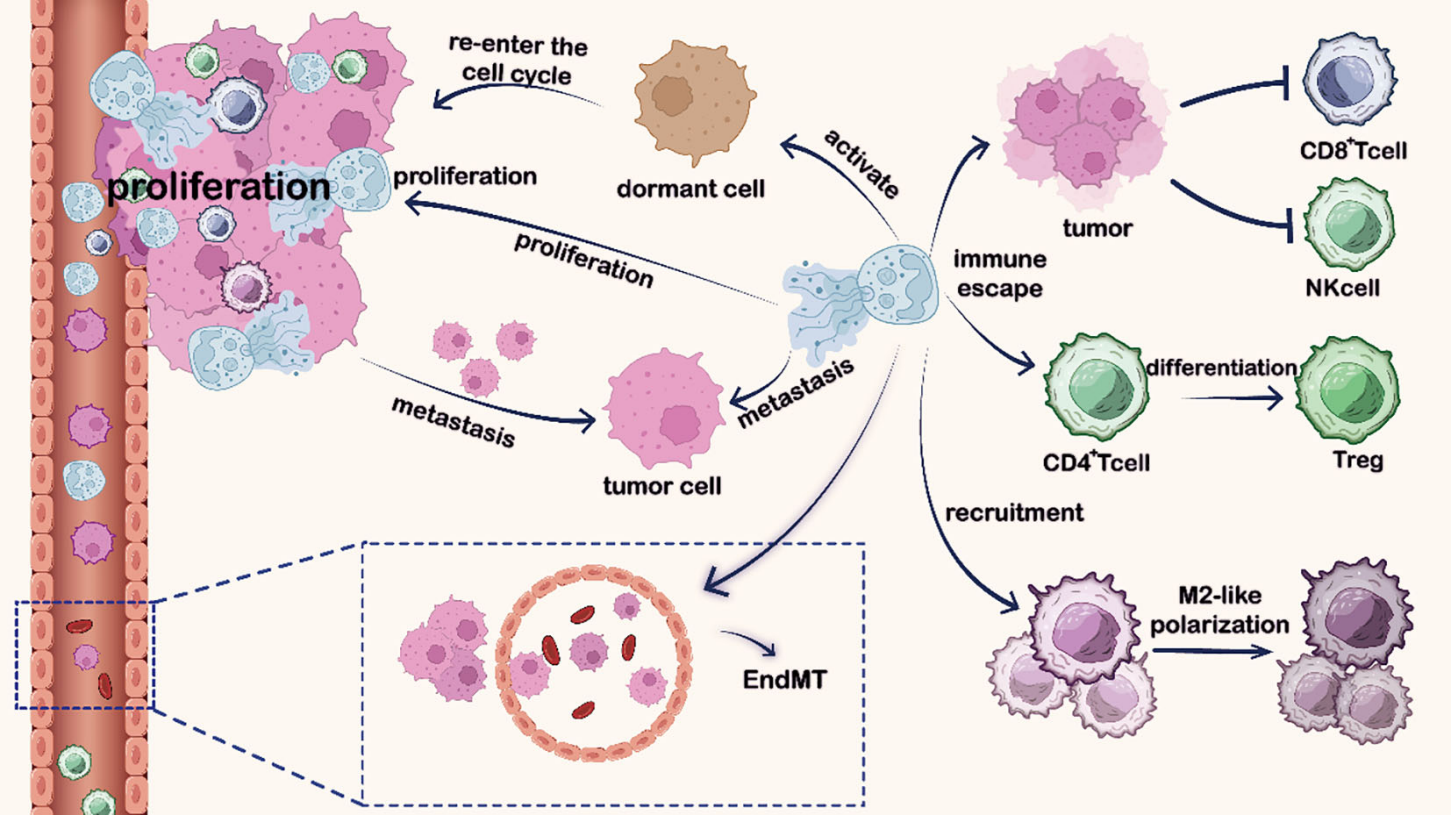
The correlated mechanisms of NETs in TME. This schematic illustrates the multifaceted role of NETs in shaping the TME. NETs promote tumor progression by facilitating immune escape through suppression of cytotoxic immune cells (e.g., CD8^+^ T cells and NK cells) and recruitment of immunosuppressive cells (e.g., Tregs). They also induce tumor cell proliferation by reactivating dormant cells and metastasis by enhancing tumor cell migration and secretion of pro-metastatic factors. Additionally, NETs drive Endothelial-to-Mesenchymal Transition (EndMT) and M2-like polarization of macrophages, further fostering a pro-tumoral TME. Together, these mechanisms highlight NETs as critical mediators of tumor aggressiveness and immunosuppression.

NETs orchestrate an immunosuppressive tumor microenvironment (TIME) through multiple mechanisms. They form a physical barrier that encapsulates tumor cells, shielding them from CD8^+^ T cells and NK cells (42). In NET−rich TIME, CD8^+^ T cells undergo exhaustion characterized by reduced secretion of IL−2, IFN−γ, and TNF−α, along with upregulation of the PD−1/PD−L1 axis (5, 66). NETs also promote differentiation of naïve CD4^+^ T cells into regulatory T cells (Tregs) via TLR4 activation and metabolic reprogramming of oxidative phosphorylation, a pathway essential for Treg immunosuppressive function (67, 68). In liver cancer models with non−alcoholic steatohepatitis, NETs co−localize with Tregs, and the NET marker H3cit correlates with FoxP3 expression (67, 69). Furthermore, NETs inhibit NK cell migration and cytotoxicity (42, 70); mechanisms include platelet activation via TGF−β and MMP−9−mediated NK cell dysfunction (71, 72). NET−derived arginase depletes local arginine, an amino acid critical for T−cell activation and proliferation, further weakening immune surveillance (73, 74). Additionally, NETs influence macrophage polarization: although limited evidence exists in tumors, NETs have been shown to drive M2−like polarization of tumor−associated macrophages (TAMs) via TREM1, and M2−type TAMs secrete VEGF and TGF−β to foster tumor growth and angiogenesis (75, 76). A comprehensive illustration of these pro−tumoral mechanisms is provided in **Figure 2**.

## 3 Regulation of NETs in the TME

**Figure 3:**
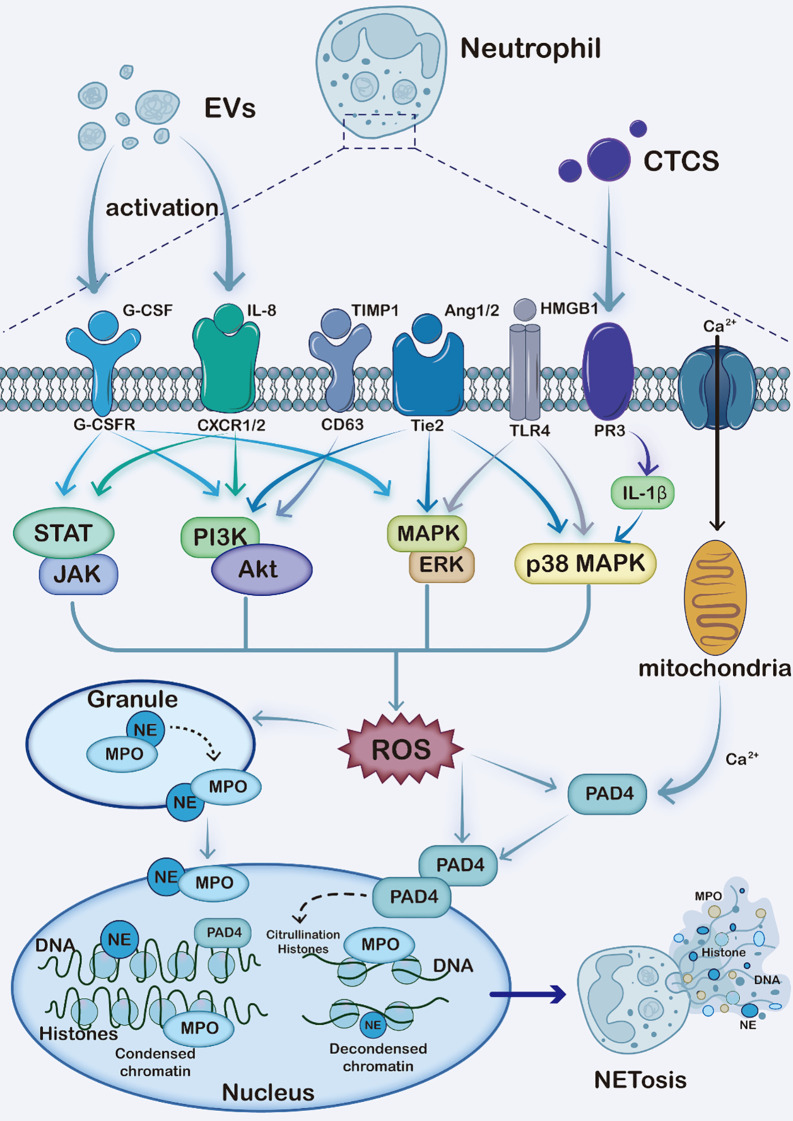
Tumor cell-secreted factors promote NET formation. Neutrophils are activated by Tumor cell-secreted factors (G-CSF, IL-8, TIMP1, Ang1/2, HMGB1 and CTCS) followed by activation of various pathways, including STAT/JAK, PI3K/Akt, MAPK/ERK, and p38MAPK signals. The endoplasmic calcium in the cytoplasm then phosphorylates NADPH Oxidase 2 (NOX2), thereby driving the production of ROS. Subsequently, NE and MPO stored in cytoplasmic granules translocate into the nucleus and contribute to chromatin decondensation with the assistance of calcium-dependent PAD4, which citrullinates histones. Decondensed chromatin mixed with granule proteins is first released into the cytoplasm and then out of the cell membrane, and forms NETs.

The tumor microenvironment (TME) provides multiple signals that promote NET formation, as illustrated in **Figure 3**. These include tumor−secreted cytokines, damage−associated molecular patterns (DAMPs), growth factors, protein regulators, extracellular vesicles, as well as hypoxia and inflammatory conditions. A summary of these inducing factors is presented in **Table 1**.

**Table 1:** Summary of NETs inducing factors and targets of action.

Predisposing factor	Sources	Targets	Functionality	Bibliography
IL-8	Tumor cells/tumor-associated fibroblasts (CAFs)	CXCR1/2-PI3K/Akt/MAPK	Recruitment of neutrophils and activation of ROS-dependent NETosis; positively associated with tumor metastasis	(42–45, 55)
IL-1β	Tumor-associated macrophages (TAMs)	TLR4/NF-κB	Promotes the formation of NETs and participates in the regulation of the inflammatory microenvironment	(19, 77)
TNF-α	Tumor-associated macrophages (TAMs)	TNFR-MAPK/NF-κB	Triggers neutrophil activation and enhances NETosis	(19, 77)
G-CSF	Tumor cells/tumor-associated immune cells	G-CSF-JAK/PI3K/MAPK	Promotes neutrophil generation and activation, induces NETosis; enhances tumor invasiveness	(56, 57, 78)
HMGB1	Necrosis of tumor cells/damaged tissue	TLR4/9-p38 MAPK/ERK/COX2	Activates ROS generation and histone citrullination via TLR signaling to drive NETosis; promotes metastasis	(79–82)
TGF-β	Tumor cells/immunosuppressive cells	Smad 2/3-LIF axis	Upregulation of LIF expression induces NETosis; associated with peritoneal metastasis	(83)
Angiopoietin (Ang1/2)	Tumor vascular endothelial cells	Tie2-MEK/p38 MAPK/PI3K	Activates NADPH oxidase and PAD4 to promote ROS-dependent NETosis enhances pro-angiogenic activity	(84–86)
VEGF	Tumor-associated endothelial cells	VEGF receptor	Promotes neovascularization and indirectly affects NET formation in TME (mechanism yet to be clarified)	(87)
Protease Cathepsin C(CTSC)	Tumor cell	Neutrophil Surface Receptor	Regulation of neutrophil recruitment and NET formation to promote breast-to-lung metastasis	(48)
TIMP-1	Tumor cell	CD63-ERK	Triggering NETosis through ERK signaling promotes pancreatic cancer progression	(88)
Haptoglobin (HP)	Tumor cells (ENPP1hi CTC)	ENPP1/HP axis	Promoting NET formation and inducing PMN-MDSC migration and invasion promotes breast cancer recurrence *in situ* through a synergistic immunosuppressive network	(89)
Extracellular vesicles (EVs)	Tumor cell	KRAS-IL-8 access road/TLR4	Carrying KRAS proteins activates IL-8 secretion or induces NETosis directly through TLR4	(90, 91)
Signs of hypoxia	Tumor-associated hypoxic regional cells	HIF-1α-NADPH oxidase	Up-regulation of NETs-related gene expression promotes pre-metastatic microenvironment formation	(80, 92)
HBV	Infections	RAGE/TLR4-ROS	Leads to the formation of abundant NETs and promotes the growth and metastasis of HCC cells	(93)
Inflammatory factors (LPS/Smoking)	LPS-activated macrophages/environmentally stimulated epithelial cells	TLR4-β1 integrin/FAK	Breaking tumor cell dormancy and activating integrin signaling to promote metastasis	(21, 22, 94)
β amyloid	Cancer-associated fibroblasts (CAFs)	CD11b-ROS	Activation of ROS-dependent NETosis via CD11b establishes a pro-metastatic positive feedback loop	(95)
IL-17	Th17 cells	IL-17/IL-17R	Recruits neutrophils and triggers NETosis, reduces CD8+T cell recruitment and activation to maintain immunosuppression	(96)
Inorganic polyphosphates (poly P)	CD68+ mast cells	TLR4/integrin signaling pathway	Activation of neutrophil ROS-dependent NETosis; promotion of pro-inflammatory and pro-metastatic responses in the colorectal cancer microenvironment	(97)
Collagen type 1 (Col1)	Cancer-associated fibroblasts (CAFs)	Col1-DDR1-NF-κB-CXCL8 axis	Stimulation of tumor CXCL8 secretion through the Col1-DDR1-NF-κB axis recruits neutrophils and initiates NETs	(12)
ECM degradation products	Proteases released by NETs (MMP2,9)	Integrin-cytoskeleton signaling	Degradation of ECMs (e.g., laminin, TSP-1), release of pro-metastatic epitopes	(93)

### 3.1 Tumor−secreted cytokines and DAMPs

Tumor cells actively secrete cytokines that drive NETosis. Interleukin−8 (IL−8) binds to CXCR1/2 on neutrophils, activating PI3K/Akt and MAPK/ERK pathways to generate ROS and promote NET release (42–44, 55); IL−8 levels correlate with NET abundance in bladder cancer, non−small cell lung cancer, and melanoma (45). Granulocyte colony−stimulating factor (G−CSF) mobilizes bone marrow neutrophils and initiates NETosis through JAK/STAT, PI3K/Akt, and MAPK/ERK pathways upon binding to G−CSFR (98); G−CSF−enriched conditions facilitate metastatic colonization in breast and ovarian cancer models (99, 100).

Damage−associated molecular patterns (DAMPs), particularly high mobility group box 1 (HMGB1), are released from necrotic or stressed tumor cells. HMGB1 induces NETosis through TLR4/9−mediated p38 MAPK/ERK signaling, and blockade of TLR4/9 inhibits NET−driven metastasis (79–81). HMGB1 also enhances neutrophil recruitment and pro−inflammatory cytokine production, forming a positive feedback loop (82).

### 3.2 Growth factors and other protein regulators

Several growth factors also contribute to NET formation. TGF−β increases LIF expression via Smad2/3 activation, leading to NET release and peritoneal metastasis in gastric cancer (83). Angiopoietin−1 and angiopoietin−2 (Ang1/2) bind to the Tie2 receptor and induce NETosis through MEK, p38 MAPK, and PI3K pathways, with Ang1 additionally supporting IL−8 synthesis (84–86).

Other tumor−derived regulators include cathepsin C (CTSC), which enhances breast−to−lung metastasis by promoting neutrophil recruitment and NET formation (48); TIMP−1, which triggers NETosis via CD63−ERK signaling in pancreatic cancer (88); and haptoglobin (HP) secreted by ENPP1−high CTCs, which induces NETs and promotes breast cancer recurrence through an immunosuppressive network (89).

### 3.3 Hypoxia, inflammation, and stromal influences

Hypoxia, a hallmark of the TME exacerbated by radiotherapy−induced vascular damage, activates HIF−1α in neutrophils and upregulates genes essential for NETosis; hypoxia−induced NETs then enhance tumor progression via TLR4/9 signaling (80, 92). Inflammatory stimuli such as cigarette smoke or LPS trigger NET deposition in premetastatic niches, disrupting tumor cell dormancy and facilitating lung metastasis (21). Postoperative abdominal infections similarly stimulate NET release, which captures free gastric cancer cells and promotes metastatic spread (94).

Stromal cells also regulate NET formation. Cancer−associated fibroblasts (CAFs) secrete β−amyloid, which activates CD11b on neutrophils and induces ROS−dependent NETosis, establishing a pro−metastatic feedback loop (95). A comprehensive summary of these NET−inducing factors is provided in **Table 1**.

## 4 Interactions between NETs, microenvironment and microbial metabolites in tumor radiotherapy

### 4.1 Role of radiation in tumor radiotherapy on the tumor microenvironment and the role of NETs

#### 4.1.1 Effects of radiotherapy on the TME

Radiotherapy induces DNA damage (double-strand breaks, single-strand breaks, chromosomal aberrations) in cancer cells and modulates the tumor microenvironment, impacting both vasculature and immune cells (101–103).

In the vascular system, irradiation causes endothelial cell dysfunction, senescence, and structural damage, leading to inflammation, fibrosis, and oxidative stress (6, 7, 104). This enhances tumor hypoxia, activating HIF-1α, which promotes radioprotection of tumor vasculature, stimulates VEGF-A-mediated angiogenesis, and induces immunosuppression by triggering effector CD8^+^ T-cell apoptosis (105–107). While combining irradiation with HIF-1α inhibitors or VEGF-A blockers is theoretically promising, specific HIF-1α inhibitors remain unapplied clinically (108, 109).

In the immune microenvironment, radiotherapy exerts dual effects: immune stimulatory actions include releasing damage-associated molecular patterns (DAMPs, e.g., ATP, HMGB-1) to activate immune surveillance via pattern recognition receptors (PRRs) (101), up-regulating MHC molecules for enhanced antigen presentation (110), increasing NK cell cytotoxicity, promoting CD8^+^T-cell infiltration and M1 macrophage accumulation (106, 111, 112), and triggering type I interferon expression through the STING pathway (113). Immunosuppressive effects involve up-regulating PD-L1 via type I/II interferons, activating TGF-β to reduce CD8^+^ T-cell cytotoxicity and promote Treg differentiation, inducing neutrophil N2 polarization, and expanding immunosuppressive cells (MDSCs, Tregs), which collectively hinder anti-tumor immune responses (114–116).

The complex balance between pro- and anti-immunogenic effects of radiotherapy, along with unclear impacts of radiation dose on immune function and tumor angiogenesis, highlights the need for deeper mechanistic insights to optimize radiotherapy and prevent recurrence.

#### 4.1.2 Role of radiotherapy on NETs

In response to radiotherapy, neutrophils are recruited and form NETs in bladder tumors. Teijeira et al. demonstrated that low-dose ionizing radiation (0.5–1 Gy) induces the formation of NETs. Specifically, ionization of oxygen molecules can trigger NETosis by activating NADPH oxidase. Additionally, NETosis is further stimulated through an autocrine IL-8-dependent mechanism (117). A related study indicated that circulating breast cancer cells overexpress ENPP1 or CD203a after radiotherapy, which increases the expression of Haptoglobin. Secreted haptoglobin promote neutrophil infiltration and NETosis via overexpression of CCR2 (89). Furthermore, Shinde-Jadhav et al. (12) elucidated the mechanism linking radiotherapy to NET formation. They found that radiotherapy induces the release of HMGB1 from bladder cancer cells, which drives the formation of NETs by activating the TLR4 signaling pathway. Following irradiation, NETs form a barrier at the tumor/stroma interface and may promote tumor radioresistance by preventing intratumoral CD8^+^ T cell infiltration post-radiotherapy (12).

### 4.2 Association of microbial metabolites with NETs and the tumor radiotherapy microenvironment

#### 4.2.1 Impact of microbial metabolites on the TME

Microorganisms colonize multiple anatomical sites of the host, and their metabolomic products are key modulators of tumorigenesis, TME remodeling, and therapeutic response (118–121). During tumor progression, intratumoral and host-gut microbial metabolites accumulate in the TME, act as ligands for pattern recognition receptors (PRRs) or modulate intracellular signaling pathway activity, and thereby reprogram the immune, vascular, and stromal components of the TME (121). Short-chain fatty acids (SCFAs, e.g., butyrate, acetate, propionate) are the most well-characterized microbial metabolites in oncology, exerting context-dependent dual effects on the TME. On the anti-tumoral side, SCFAs target cancer driver genes and signaling pathways to maintain cellular homeostasis: acetic acid binds to hepatic GPR43 to inhibit the JAK1/STAT3 pathway and suppress hepatocellular carcinoma progression (122); butyrate inhibits histone deacetylase (HDAC) to upregulate CD25 expression and enhance the cytotoxicity of CD8^+^ T cells in pancreatic cancer and melanoma (123). On the pro-tumor side, SCFAs remodel the immunosuppressive TME by regulating immune cell polarization and inflammatory signaling, and activate the MAPK/PI3K pathway to promote cancer cell proliferation (124, 125). Beyond SCFAs, other microbial metabolites also play critical roles in TME regulation: indole derivatives (tryptophan metabolites) activate the aryl hydrocarbon receptor (AhR) to modulate the anti-tumor immune response (125); deoxycholic acid (DCA, a bile acid metabolite) regulates stromal cell activation and tissue repair (126); propionate modulates the polarization of tumor-associated macrophages (TAMs) and the recruitment of regulatory T cells (Tregs) (127). Collectively, microbial metabolites are pleiotropic regulators of the TME, and their functional outcomes are tightly linked to the metabolite type, concentration, and the intrinsic characteristics of the TME (e.g., hypoxia, inflammation, immune cell composition). Targeting microbial metabolites or their downstream signaling holds great potential to optimize TME status and enhance radiotherapy efficacy while reducing off-target effects.

#### 4.2.2 Effects of microbial metabolites on NETs

Microbial metabolites modulate NET formation through tissue- and context-specific molecular mechanisms, with current research focusing primarily on SCFAs, and emerging evidence for tryptophan metabolites and bile acid metabolites. For SCFAs, butyrate consistently exerts an inhibitory effect on NET formation across different disease models, with a well-elucidated mechanism: butyrate produced by R. intestinalis inhibits NOX2-dependent ROS generation in neutrophils, thereby reducing NET formation, neutrophil infiltration, and inflammatory responses in abdominal aortic aneurysms (128); in inflammatory bowel disease (IBD), butyrate suppresses neutrophil ROS production, NET release, and pro-inflammatory cytokine secretion to alleviate mucosal inflammation (129); in calculous cholecystitis, butyrate reduces macrophage-derived CXCL16 secretion to inhibit neutrophil migration and subsequent NET formation (130).

In contrast, acetate exerts bidirectional effects on NETosis that depend on cell type and concentration, which explains the contradictory findings in previous studies: in primary human peripheral blood neutrophils, physiological concentrations of acetate moderately inhibit NET formation through an uncharacterized NOX-independent mechanism (131); in DMSO-differentiated neutrophil-like HL-60 cells, supraphysiological concentrations of sodium acetate enhance NETosis via the histone acetylation pathway, a process that is independent of NOX-derived ROS (132). This discrepancy is likely attributed to two key factors: (1) cell type heterogeneity: HL-60 cells are immortalized myeloid leukemia cells with abnormal epigenetic and signaling regulation, which cannot fully recapitulate the biological behavior of primary neutrophils; (2) concentration dependence: physiological acetate concentrations (maintained by microbial metabolism) regulate neutrophil homeostasis and inhibit excessive NET formation, while supraphysiological concentrations (artificially induced) disrupt histone modification balance and trigger aberrant NET release. Additionally, the TME context (e.g., hypoxia, high inflammatory factor levels) may further modify acetate’s effect on NETs by regulating neutrophil metabolic status and receptor expression.

For non-SCFA microbial metabolites, preliminary studies have revealed their regulatory potential on NETs: propionate (a SCFA) inhibits systemic inflammation and indirectly reduces NET formation by modulating neutrophil activation (127); tryptophan metabolites (e.g., kynurenine) may regulate NETosis via the AhR pathway (unpublished data), and bile acid metabolites (e.g., DCA) reduce radiation-induced inflammatory NET formation by promoting endothelial cell repair (126). However, the direct effects and molecular mechanisms of these non-SCFA metabolites on NET formation in the TME remain largely unexplored and require further *in vivo* and *in vitro* validation in cancer models.

#### 4.2.3 Effects of microbial metabolites on radiotherapy and the unifying microbial metabolite-NETs-radiotherapy axis

Recent studies have established a critical regulatory role of gut microbiome-derived metabolites in radiotherapy efficacy, which is closely linked to their modulation of NET formation and anti-tumor immunity (127, 133, 134). A unifying mechanistic model for the microbial metabolite-NETs-radiotherapy axis is proposed here (**Figure 1**), which fundamentally includes two bidirectional regulatory loops and is governed by the type and context of microbial metabolites: (1). Anti-tumor loop (microbial metabolites → NET inhibition → radiotherapy sensitization): Depletion of vancomycin-sensitive gut bacteria eliminates SCFAs (butyrate, propionate) that induce immunosuppression and NET formation, thereby reducing the accumulation of immunosuppressive NETs in the TME, enhancing dendritic cell (DC) antigen presentation, promoting tumor-specific CD8^+^ T cell activation, and significantly improving radiotherapy efficacy (133). Conversely, oral supplementation of these bacteria increases butyrate levels in the TME, which inhibits radiotherapy-induced type I interferon (IFN) responses and reduces anti-tumor immunity (134)—this pro-tumor effect is partially mediated by butyrate’s indirect regulation of NET-associated T cell exhaustion. Additionally, butyrate’s direct inhibition of pro-tumor NET formation (e.g., NET-mediated CD8^+^ T cell exclusion, circulating tumor cell (CTC) encapsulation) can sensitize tumors to radiotherapy by restoring the anti-tumor immune microenvironment. (2). Radioprotective loop (microbial metabolites → NET modulation → reduced radiation toxicity): Microbial metabolites (SCFAs, propionate, tryptophan metabolites, DCA) mitigate radiation-induced normal tissue damage by modulating NET formation and inflammatory responses (126, 127): SCFAs promote the repair of hematopoietic and intestinal epithelial cells, suppress systemic inflammation by reducing pro-inflammatory cytokine secretion and inducing IL-10, and their radioprotective effect is associated with the inhibition of excessive NET formation in normal tissues (127); DCA accelerates the healing of radiation-induced skin injuries by inhibiting inflammatory NET deposition and promoting tissue repair (126). This loop is independent of the anti-tumor effect and provides a strategy to separate radiotherapy efficacy from normal tissue toxicity by targeting microbial metabolite-mediated NET modulation.

Notably, the balance between these two loops is tightly regulated by the TME and microbial metabolite profile: in the tumor core (hypoxic, high pro-inflammatory factor levels), microbial metabolites that inhibit pro-tumoral NET formation (e.g., physiological butyrate) dominate the anti-tumoral loop and enhance radiotherapy efficacy; in normal tissues (normoxic, low inflammation), metabolites that inhibit inflammatory NET formation (e.g., propionate, DCA) dominate the radioprotective loop and reduce off-target damage. In addition, concentration-dependent effects of SCFAs (e.g., acetate) are a key variable of this axis: supraphysiological acetate levels may induce aberrant NET formation in the TME, leading to radioresistance and distant metastasis, while physiological acetate levels inhibit excessive NET formation and enhance radiotherapy sensitization.

Translationally, targeting the microbial metabolite-NETs-radiotherapy axis holds great promise for personalized radiotherapy: (1) microbiome modulation: fecal microbiota transplantation (FMT) or probiotic supplementation (e.g., R. intestinalis that produces butyrate) can optimize the microbial metabolite profile to inhibit pro-tumor NET formation and enhance radiotherapy efficacy; (2) metabolite targeted intervention: combined administration of physiological concentrations of butyrate/propionate with radiotherapy can sensitize tumors while reducing radiation toxicity; (3) co-targeting of metabolites and NETs: combining microbial metabolite modulators with NET inhibitors (e.g., DNase I, GSK484) can block the pro-tumor NET pathway and amplify the anti-tumor effect of radiotherapy. However, the clinical application of this axis still faces challenges, such as the lack of biomarkers for predicting the microbial metabolite-NET profile of individual patients, and the need to clarify the tissue-specific effects of microbial metabolites on NETs. Future studies should focus on the dynamic changes of the microbial metabolite-NETs axis during radiotherapy and develop personalized intervention strategies based on patient-specific microbiome and TME characteristics.

## 5 Clinical Implications: NETs in radiotherapy – friend or foe?

**Figure 4:**
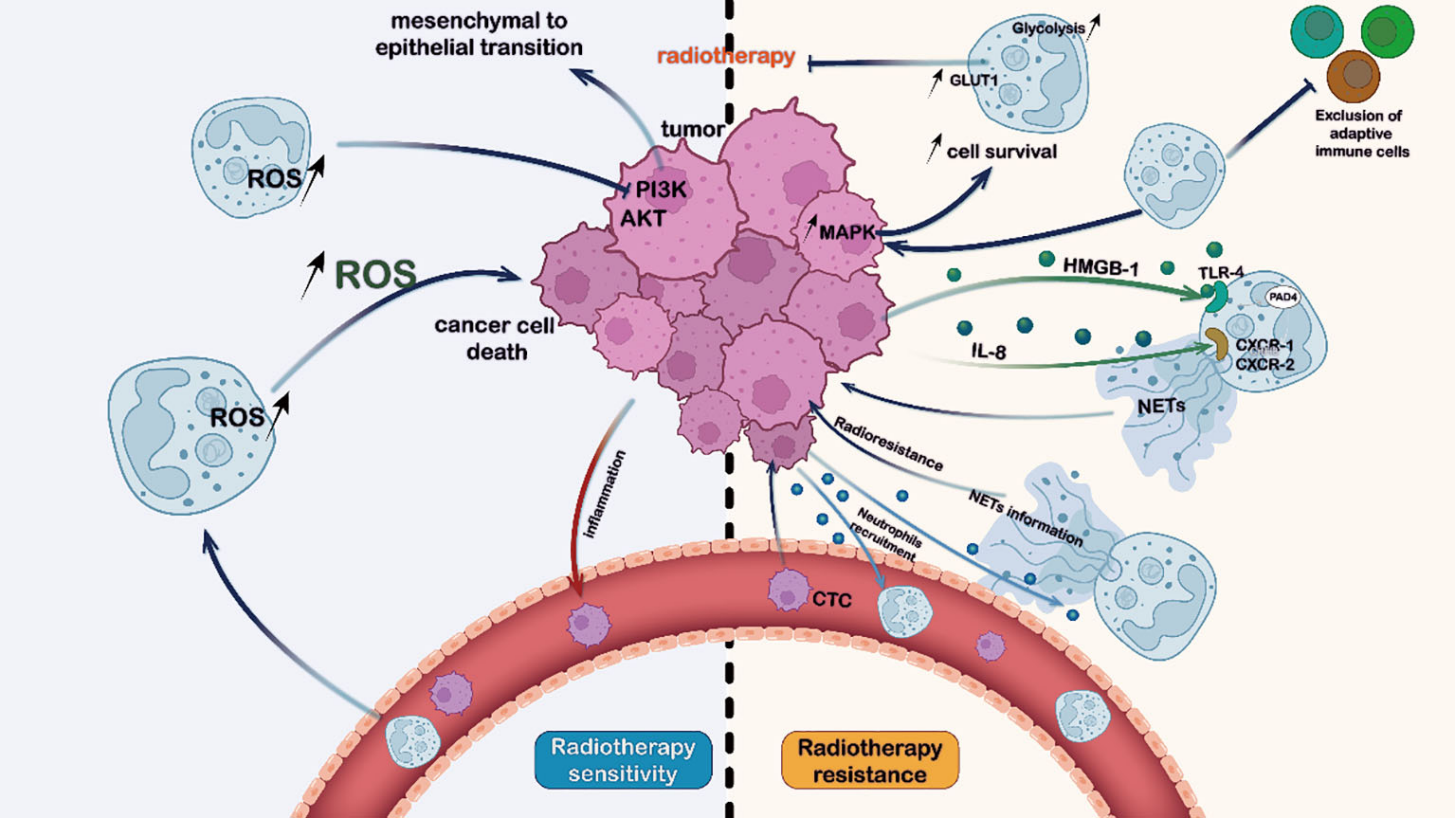
Impact of neutrophils and neutrophil extracellular traps (NETs) on the response to radiotherapy. Left panel (therapy-enhancing, anti-tumoral effects): Radiotherapy can recruit and polarize neutrophils toward an anti-tumoral (N1) phenotype. These activated neutrophils generate high levels of reactive oxygen species (ROS), directly inducing tumor cell apoptosis (78). Furthermore, they can promote the activation and recruitment of tumor-specific T cells, thereby fostering a potent anti-tumor immune response that synergizes with radiotherapy (138, 139). Right panel (therapy-limiting, pro-tumoral effects): Radiotherapy can stimulate the release of NETs, driven by factors such as HMGB1 and IL-8 (12, 117). These NETs form a physical barrier at the tumor-stroma interface, which sequesters cytotoxic CD8+ T cells and prevents their infiltration into the tumor, leading to radioresistance (12). Concurrently, NETs can encapsulate circulating tumor cells (CTCs), promoting their survival and facilitating distant metastasis (42, 117).

NETs have been extensively studied and confirmed to bi-directionally regulate tumor development, exhibiting dual functional properties-both pro-tumoral and anti-tumoral (135). A recent review suggests that neutrophils and NETs also play a dual role in influencing the efficacy of anticancer therapies (136). However, our understanding of the involvement of NETs in radiotherapy remains incomplete and is characterized by conflicting results (**Figure 4**).

### 5.1 Pro-tumoral effects: NETs promote radioresistance

Studies link NETs to radioresistance, with enriched NETs in radioresistant tumors correlating with shorter patient survival (12). In a bladder cancer model, RT increased NET deposition in the TIME, while DNase I (degrading NETs-DNA) or NE inhibitors sensitized tumors to RT, slowing growth and improving T-cell infiltration (12). Clinically, high tumor NET expression predicts poor prognosis and RT insensitivity in bladder cancer patients. PAD4 inhibitor GSK484 and HMGB1 inhibitor glycyrrhizin also reduce NET formation and enhance RT response (12, 137).

In breast cancer, ENPP1-overexpressing CTCs drive recurrence via PMN-MDSC-mediated NET formation, with ENPP1 and NET levels elevated in recurrent tumors. Inhibiting ENPP1 or NETs prolongs recurrence-free survival, highlighting their role in self-seeding metastasis (89).

Existing studies have shown that NETs are closely related to tumor therapy resistance, but the role of NETs in radioresistance and the mechanistic role of NETs are still unclear. The study by Shinde-Jadhav et al. (12) suggests a possible mechanism by which NETs contribute to radioresistance. By immunofluorescence analysis, a physical barrier of NETs formed at the MB49 tumor-stroma interface after radiation, isolating CD8^+^ T cells from the periphery, revealing that this barrier mediates radiotherapy resistance by limiting cytotoxic T-cell infiltration. In addition, Teijeira et al. (117) found that contralateral breast radiotherapy or intravenous administration of radiation-induced NETs significantly increased the pulmonary metastatic load through a model of spontaneous metastatic breast cancer, further confirming that radiotherapy-induced NETs may be involved in mediating radioresistance through a mechanism that promotes distant metastasis of the tumor.

### 5.2 Anti-tumoral effects: NETs may enhance radiotherapy efficacy

Radiotherapy can trigger aseptic inflammation, prompting rapid and transient infiltration of tumor tissue by neutrophils. These infiltrating neutrophils produce large amounts of ROS, which can induce apoptosis in tumor cells, thereby inhibiting tumor growth and enhancing the efficacy of radiotherapy. In the Lewis lung cancer (LLC) model, radiotherapy activates neutrophil recruitment and polarizes them to an anti-tumoral phenotype. High ROS levels reverse epithelial-mesenchymal transition (EMT) in LLC cells by inhibiting the PI3K and protein kinase B (Akt) signaling pathways, which induces apoptosis of the tumor cells, and enhances the therapeutic effect of radiotherapy (78). In addition, when radiotherapy is combined with G-CSF, neutrophils are activated, which can promote the activation and recruitment of tumor-specific T cells to the tumor site (138). T cells can recognize and attack tumor cells, enhance the body’s anti-tumor immune response, and thus improve the therapeutic effect of radiotherapy (138). In a mouse model of lymphoma, radiotherapy combined with complement inhibition therapy enhanced the therapeutic effect by promoting tumor cell apoptosis, inflammatory response, and early influx of neutrophils; whereas removal of neutrophils completely eliminated the enhancing effect of complement inhibition on the efficacy of radiotherapy, which suggests that neutrophils play a key role in promoting the anti-tumor immune response to radiotherapy (139).

While less studied, NETs likely collaborate with infiltrating neutrophils in the RT-induced sterile inflammatory microenvironment. NETs may migrate to tumor sites, synergizing with neutrophils to generate ROS and regulating neutrophil polarization and T-cell interactions to enhance anti-tumor immunity. Their potential role in boosting RT efficacy, inferred from the functions of neutrophils and the biological properties of NETs, warrants further exploration to uncover new strategies for improving radiotherapy outcomes.

Radiotherapy induces a complex response involving neutrophils and neutrophil extracellular traps (NETs) that can paradoxically either enhance or diminish treatment efficacy.

### 5.3 Contextual determinants of NETs’ dual role in radiotherapy

The coexistence of pro- and anti-tumoral NET responses to radiotherapy raises a critical question: what determines whether NETs act as “friends” or “foes”? Emerging evidence points to several interdependent determinants.

Radiation dose may serve as an initial trigger. Teijeira et al. demonstrated that low-dose (0.5–1 Gy) radiation elicits NET formation via oxidative stress, leading to immunosuppressive effects (117). In contrast, Shinde-Jadhav et al. employed conventional doses (2–10 Gy) and observed NETs forming a physical barrier that excluded CD8^+^ T cells and promoted radioresistance (12). Higher doses induced more rapid NET formation, suggesting different doses may induce qualitatively distinct NETs.

This qualitative difference likely involves the mode of NETosis. Suicidal NETosis releases nuclear DNA and granular proteins that degrade ECM and awaken dormant cancer cells. Vital NETosis preserves neutrophil viability and may produce NETs that primarily modulate immune responses (21, 24). Different stimuli preferentially trigger distinct pathways—PMA and ionomycin primarily induce suicidal NETosis (140), while LPS can trigger vital NETosis, particularly in the presence of platelets (23, 39). Whether different radiation doses preferentially activate one pathway remains unknown.

The functional differences between NETosis modes likely stem from their resultant NET molecular composition—their “molecular fingerprint”. Proteomic analyses reveal that NETs contain over 5,800 proteins, with composition varying by stimulus (141), and histones within NETs carry distinct oxidative modifications depending on the inducer (142). Thus, radiotherapy-induced NETs may possess distinct “molecular fingerprints” dictated by the initiating NETosis pathway, which in turn determine their pro-tumoral versus anti-tumoral functions.

Spatiotemporal dynamics further modulate NETs’ net impact. Spatially, NETs at the tumor-stroma interface form physical barriers excluding CD8^+^ T cells (12), while intratumoral NETs may directly interact with cancer cells. Reichardt et al. showed irradiation alters neutrophil spatial organization, reducing neutrophil-rich neighborhoods correlated with improved survival (143). Temporally, an early, transient NET wave may contribute to acute inflammation, whereas late, sustained NET accumulation could promote fibrosis and resistance (12, 117).

Other factors include tumor type and TME composition (80, 92). Understanding these interdependent determinants is essential for designing rational strategies that selectively inhibit detrimental NETs while preserving beneficial effects.

## 6 Therapeutic targeting of NETs in combination with radiotherapy

Radiotherapy (RT) induces NET formation through mechanisms involving HMGB1−TLR4 signaling, ROS generation, and autocrine IL−8 loops (12, 117). Once formed, NETs contribute to radioresistance by creating a physical barrier that excludes CD8^+^ T cells and by promoting distant metastasis (12, 42, 117). Therefore, targeting NETs represents a promising strategy to improve RT outcomes. Several approaches have been directly evaluated in combination with RT, and additional candidates are rationally supported by the molecular pathways activated by irradiation. The main strategies are discussed below, and a summary is provided in **Table 2**.

**Table 2:** Potential therapeutic strategies targeting NETs to enhance radiotherapy.

Intervention strategies	Targets/mechanisms	Efficacy in radiotherapy context	Limitations	References
A. Inhibition of NET formation				
PAD4 inhibitors (GSK484)	Inhibits histone citrullination, blocks NETosis	Colorectal cancer: GSK484 increases radiosensitivity by inhibiting NET formation (137); Bladder cancer: GSK484 reduces NET deposition and enhances RT response (12)	Potential immunosuppression	(12, 137)
NE inhibitor	Inhibits neutrophil elastase	Invasive bladder cancer model: RT induced NETs via HMGB1-TLR4. NE was identified as a key NET component, and its inhibition (alongside other NET-targeting strategies) improved RT response.	Infection risk; may impair tissue repair	(144)
HMGB1 inhibitors (glycyrrhizin)	Blocks HMGB1−TLR4/9, reduces NETosis	Invasive bladder cancer model: RT (2−10 Gy) induced HMGB1 release, triggering NETosis via TLR4. Glycyrrhizin reduced NET formation and improved RT response.	Limited clinical data; potential systemic effects	(12)
ENPP1 inhibitor	Inhibits ENPP1−CD203a axis, reduces NET formation	Breast cancer postoperative model: After RT, CTCs overexpressed ENPP1/CD203a, leading to haptoglobin secretion and NETosis. ENPP1 inhibition reduced NET formation and decreased post−RT local recurrence.	May affect normal nucleotide metabolism	(89)
CXCR1/2 antagonist (reparixin)	Blocks IL−8 signaling, inhibits ROS and NETosis	Tumor model: Reparixin reduced NET deposition and improved anti−tumor immunity. Low−dose RT induces IL−8−dependent NET formation, making CXCR1/2 blockade a rational radiosensitizer.	May inhibit anti−tumoral neutrophils	(42, 117)
B. Disruption of existing NETs				
DNase I	Degrades NET DNA backbone	Invasive bladder cancer model: DNase I enhances RT sensitivity and improves CD8^+^ T-cell infiltration by eliminating NET physical barrier.	Short half−life; requires frequent dosing	(12)
C. Combination with immune checkpoint blockade				
Anti−PD−1/PD−L1	Blocks PD−1/PD−L1 axis	Cirrhotic HCC model: DDR1 inhibitor−mediated NET clearance restored anti−PD−1 efficacy. RT induces PD−L1 expression, supporting triple combination (RT + NET inhibitor + anti−PD−1).	Immune−related adverse events	(5, 66, 145)

Other strategies targeting NETs have shown promise in preclinical cancer models but lack direct evidence in the context of radiotherapy; these are discussed in the main text (section 3).

### 6.1 Inhibiting NET formation

Preclinical studies have tested several inhibitors alongside RT. The PAD4 inhibitor GSK484 blocks histone citrullination, a non−reversible step in NETosis, and enhances radiosensitivity in colorectal cancer models (137). The HMGB1 inhibitor glycyrrhizin reduces NET formation by blocking the HMGB1−TLR4 pathway and improves RT response in invasive bladder cancer (12). ENPP1 inhibitors target the ENPP1−CD203a axis, reduce NET formation, and lower the risk of post−RT recurrence in breast cancer (89). CXCR1/2 antagonists such as reparixin decrease NET deposition and enhance anti−tumor immunity by blocking the IL−8−CXCR1/2 axis that drives NET formation (42). Low−dose RT (0.5−1 Gy) has been shown to induce IL−8−dependent NET release (117), providing a strong mechanistic rationale for combining CXCR1/2 blockade with radiotherapy to overcome the NET−mediated barrier that limits T−cell infiltration. Although not all of these agents have been combined with RT in every tumor type, the bladder cancer and breast cancer models provide direct proof−of−concept for radiosensitization.

### 6.2 Degrading existing NETs

An alternative to preventing NET formation is to eliminate NETs after they are released. DNase I cleaves the DNA backbone of NETs and has been successfully combined with RT. In a bladder cancer model, DNase I treatment delayed tumor growth and increased intratumoral CD8^+^ T cell infiltration (12). In metastatic breast cancer models, DNase I prevented NET−induced distant metastasis (63). These findings support the clinical evaluation of DNase I as an adjunct to RT. Heparin can also disrupt NETs by binding histones, but its utility in RT is limited by bleeding risk and lack of direct combination data (146).

### 6.3 Combination with immune checkpoint inhibitors

NETs upregulate PD−L1 and promote T−cell exhaustion, providing a strong rationale for combining NET−directed therapies with immune checkpoint blockade. RT itself induces PD−L1 expression, suggesting triple combinations could be particularly effective. In a cirrhotic hepatocellular carcinoma model, DDR1 inhibitor−mediated NET clearance restored the efficacy of anti−PD−1 therapy (145). ARG1 monoclonal antibodies neutralize NET−associated arginase, restoring arginine levels in the microenvironment and enhancing anti−PD−1 efficacy in pancreatic cancer, a strategy that could be combined with RT (147). Anti−G−CSF antibodies reduce neutrophil mobilization and NET release; RT is known to increase G−CSF levels, making this combination intriguing (46).

### 6.4 Future challenges

Despite promising preclinical data, several challenges remain. The functional heterogeneity of NETs—depending on tumor type, radiation dose, and the mode of NETosis (suicidal versus vital)—suggests that patient stratification will be essential. Systemic inhibition of NET formation may increase infection risk or impair wound healing, as NETs play a protective role in host defense (148, 149). Localized or transient inhibition strategies, along with biomarker−driven patient selection, should be prioritized in future trial design.

## 7 Summary and future prospects

The dual role of neutrophil extracellular traps (NETs) in radiotherapy has garnered considerable attention, yet clinical translation remains challenging due to their functional heterogeneity within the tumor microenvironment (12, 117). This review has systematically examined the mechanisms by which NETs can both enhance and limit radiotherapy efficacy.

Accumulating evidence indicates that the net impact of NETs is context-dependent, governed by multiple interrelated factors including radiation dose, mode of NETosis, molecular composition, and spatiotemporal dynamics. Nevertheless, several critical questions remain unresolved.

First, whether suicidal versus vital NETosis yield functionally distinct NETs in the radiotherapy context—and whether different radiation doses preferentially activate one pathway—remains to be elucidated (2, 4, 5, 11). Second, although proteomic analyses demonstrate that NET molecular composition varies by stimulus (141, 142) how specific molecular “fingerprints” translate into pro- versus anti-tumoral functions requires systematic investigation. Third, the factors governing NET spatiotemporal dynamics—intratumoral versus stromal localization and the transition from early to late post-RT accumulation—require further study (12, 15, 143).

Addressing these knowledge gaps will require further research to better understand how different NET subtypes function in the tumor microenvironment. By identifying which NETs contribute to radiotherapy resistance and which enhance treatment response, we can develop more targeted therapeutic strategies—such as PAD4 inhibitors, DNase therapy, or ROS scavengers (12, 137, 150). Ultimately, a clearer understanding of NET biology will enable clinicians to optimize radiotherapy by selectively inhibiting harmful NETs while preserving beneficial ones.
